# HVEM and CD160: Regulators of Immunopathology During Malaria Blood-Stage

**DOI:** 10.3389/fimmu.2018.02611

**Published:** 2018-11-13

**Authors:** Franziska Muscate, Nadine Stetter, Christoph Schramm, Julian Schulze zur Wiesch, Lidia Bosurgi, Thomas Jacobs

**Affiliations:** ^1^Protozoa Immunology, Bernhard Nocht Institute for Tropical Medicine, Hamburg, Germany; ^2^1st Department of Medicine, University Medical Centre Hamburg-Eppendorf, Hamburg, Germany; ^3^Martin Zeitz Centre for Rare Diseases, University Medical Centre Hamburg-Eppendorf, Hamburg, Germany

**Keywords:** cerebral malaria, CD8 T cells, co-inhibitory receptors, HVEM, CD160

## Abstract

CD8^+^ T cells are key players during infection with the malaria parasite *Plasmodium berghei* ANKA (PbA). While they cannot provide protection against blood-stage parasites, they can cause immunopathology, thus leading to the severe manifestation of cerebral malaria. Hence, the tight control of CD8^+^ T cell function is key in order to prevent fatal outcomes. One major mechanism to control CD8^+^ T cell activation, proliferation and effector function is the integration of co-inhibitory and co-stimulatory signals. In this study, we show that one such pathway, the HVEM-CD160 axis, significantly impacts CD8^+^ T cell regulation and thereby the incidence of cerebral malaria. Here, we show that the co-stimulatory molecule HVEM is indeed required to maintain CD8^+^ T effector populations during infection. Additionally, by generating a CD160^−/−^ mouse line, we observe that the HVEM ligand CD160 counterbalances stimulatory signals in highly activated and cytotoxic CD8^+^ T effector cells, thereby restricting immunopathology. Importantly, CD160 is also induced on cytotoxic CD8^+^ T cells during acute *Plasmodium falciparum* malaria in humans. In conclusion, CD160 is specifically expressed on highly activated CD8^+^ T effector cells that are harmful during the blood-stage of malaria.

## Introduction

The inflammatory response to blood-stage malaria is characterized by a strong Th1 polarization and T cell induced immunopathology. It has been shown that when C57BL/6J mice are infected with the parasite *Plasmodium berghei* ANKA (PbA), cytotoxic CD8^+^ T cells do not contribute to the elimination of the parasite during blood-stage, but rather cause the disruption of the blood-brain barrier. *Plasmodial* antigens can indeed be cross-presented on activated brain endothelial cells (1) leading to the release of cytotoxic molecules and pro-inflammatory cytokines such as granzymes and IFNγ by T cells (2–5). This leads to the severe manifestation of experimental cerebral malaria (ECM) (5).

T cell function is tightly controlled by the integration of co-inhibitory and co-stimulatory signals. We have shown and so have others that the co-inhibitory receptors PD-1, CTLA4 and BTLA are induced during malaria. These co-inhibitory receptors play an important role in the regulation of CD4^+^ T cell activation thus controlling immunopathology during the blood-stage (6–11). In contrast, during the liver-stage of malaria they restrict the protective function of CD8^+^ T cells (12). Of note, the control of CD8^+^ T cells during the blood-stage of the infection and ECM remains to be fully understood. Dissection of the impact of different immunomodulatory receptors in T cell regulation is essential not only for our understanding of T cell biology but also for the therapeutic use of checkpoint inhibitors. This might allow us to dampen unwanted immune responses without lowering protection and to increase protection without the risk of overwhelming inflammation. We have previously described the protective function of BTLA antagonists in experimental cerebral malaria (7), yet a deeper understanding of the HVEM-network during malaria still represents a crucial step to develop and to broaden future therapeutic approaches. Besides BTLA, CD160, LIGHT, and LTα are known HVEM-ligands. LIGHT-HVEM interaction does not impact the development of experimental cerebral malaria, while local LTα drives cerebral pathology (13, 14). HVEM is an important co-stimulatory receptor expressed by T and B cells, endothelial cells and mast cells (15–18). HVEM-signaling enhances the expansion of T cells and is required for the persistence of memory T cells in bacterial and viral infections and in experimental settings of challenge with model antigen (19–22). Still its exact function in models of parasitic infections requires further investigation. We hypothesized that during malaria, HVEM via co-stimulation of CD8^+^ T cells plays a critical role in the development of the cerebral symptoms. In addition, we also focused our attention on the HVEM-ligand CD160, which shares the binding region with BTLA. CD160 is described to be expressed by cytotoxic NK and CD8^+^ T cells, NKT cells, a minority of CD4^+^ T cells, γδ T cells, iIELs, ILC1, and mast cells (23–28). CD160 function on T cell regulation remains controversial as some reports describe CD160 to be a co-inhibitory molecule whereas others suggest that it exhibits a co-stimulatory function enhancing proliferation, inflammatory cytokine production and cytotoxic capacity (15, 29–32). In this study we addressed this unanswered question by generating CD160-deficient mice and analyzing the CD8^+^ T cell profile during experimental cerebral malaria.

Our data demonstrate that HVEM is required to stabilize CD8^+^ T effector cell populations during acute *P. berghei* ANKA (PbA) infection. Expression of CD160 is specifically induced in highly activated, cytotoxic CD8^+^ T cells concurrently with the onset of cerebral symptoms. However, an aggravated T cell mediated pathology upon infection with PbA in CD160^−/−^ mice suggests a co-inhibitory function of CD160. Importantly, we found CD160 expression by CD8^+^ T cells not only in mice infected with PbA but also in patients suffering from acute malaria.

## Materials and methods

### Mice

C57BL/6J, HVEM^−/−^ (33), CD160^−/−^ (see section Generation of CD160^−/−^ Mice), OT-1CD45.2CD90.1 and HVEM^−/−^ OT-1CD45.2 (from now on referred to without congenic markers) mice were bred in the animal facility of the Bernhard Nocht Institute for Tropical Medicine and maintained in a specific pathogen-free facility. Age-matched (7–8 weeks of age) female mice were used. All experiments with mice were approved by the City of Hamburg Office for Consumer Protection (56/13; 32/15).

### Human samples

All experiments performed with human peripheral blood were approved by the Ethics Committee of the Medical Association Hamburg. Male malaria patients at an average age of 44 (range 26–69 years) with a travel history in malaria endemic regions and confirmed *Plasmodium falciparum* positive blood smears (Ethics Approval PV4539) were recruited between October 2015 and August 2016 at the University Medical Center Hamburg-Eppendorf. The patient samples include anonymous acquired discarded tissue samples of the diagnostic lab at the Bernhard Nocht Institute for Tropical Medicine. Analysis of blood samples was performed at the day of transmission to hospital or up to three days after treatment. Five patients had a parasitemia lower than 1%, three lower than 3% and one patient showed hyperparasitemia (8%) at the transmission to hospital. Members of the Bernhard Nocht Institute for Tropical Medicine were recruited as healthy controls. Control patients with chronic hepatitis B virus infection (HBV), autoimmune liver diseases (primary biliary cholangitis (PBC), primary sclerosing cholangitis (PSC) and autoimmune hepatitis (AIH) were recruited at the liver outpatient clinics at the University Medical Center Hamburg-Eppendorf and gave written informed consent (Ethics Approval PV4081; PV5661). In total, 10 malaria patients and 9 healthy controls were included in the study. Informed consent was obtained from all individuals included.

### Flow cytometry of human cells

Peripheral blood samples were first stained for surface epitopes [αCD8 AF700 (53-6.7), αCD28 BV510 (CD28.2), αCD69 FITC (FN50), αCD160 PE-Cy7 (By55), αPD-1 PerCP-Cy5.5 (EH12.2H7) from BioLegend] including a live/dead staining reagent (LIVE/DEAD Fixable Blue Dead Cell Stain Kit for UV excitation from ThermoFisherScientific) for 30 min at 4°C. Afterwards, lymphocytes were fixed and red blood cells (RBCs) were lysed (1-step Fix/Lyse solution from eBioscience). Fixation and permeabilisation of cells was performed using the Foxp3/Transcription Factor Buffer Set (ThermoFisherScientific) according to the manual. To block unspecific binding of antibodies, cells were incubated for 10 min at 4°C with CohnII and subsequently, antibodies directed against intracellular epitopes (GzmB AF647 (GB11), CTLA-4 PE (L3D10), Ki67 AF488 (Ki-67), CD3 APC-Cy7 (HIT3a) from BioLegend, Perforin BV421 (δG9) from BD) were added and further incubated for 20 min at 4°C. Samples were recorded using the LSRII (BD) and analyzed using the FlowJo X 10.0.7r2 Treestar software. Gates were set according to fluorescence minus one (FMO) controls. Gating strategy is depicted in Supplementary Figure 5.

### Adoptive, competitive T cell transfer

CD8α^+^ T cells were isolated from total splenocytes of OT-1CD45.2CD90.1 and OT-1CD45.2xHVEM^−/−^ by MACS (CD8α^+^ T Cell Isolation Kit, mouse from Miltenyi), labeled with proliferation dyes (Cell Proliferation Dye eFluor^TM^ 450 or 670 from eBioscience) according to the manual. Both CD8^+^ T cell populations were mixed in a 1:1 ratio and transferred i.v. in C57BL/6JCD45.1 mice. Mice were infected on the same day with Pb-OVA (see 2.4). Five days after transfer and infection, blood and splenocytes were isolated and analyzed.

### Plasmodium infections

*Plasmodium berghei, Plasmodium yoelii*, and *Plasmodium berghei-OVA* (34) parasites were stored in 0.9% NaCl, 4.6% sorbitol and 35% glycerol in liquid nitrogen. In order to increase viability of the parasites, they were passaged once in C57BL/6J mice. Subsequently, fresh blood of passage mice was used to transfer 1x 10^5^ infected red blood cells/200 μl PBS i.p. in mice used for experiments.

Cerebral symptoms were scored according to the following scheme: 0 = no symptoms; 1 = decreased activity, deceleration; 2 = ataxia; 3 = weight loss≥ 20% or convulsions, strong ataxia; 4 = coma; 5 = death. If a score of ≥3 was reached, mice were euthanized in order to avoid unnecessary suffering.

### Flow cytometry of murine cells

Splenocytes were isolated as described previously (35). Blood was collected using heparinized syringes and washed in PBS. Red blood cells were lysed by incubation in lysis buffer (10 nM tris pH 7.2, 0.15 M ammonium chloride). Brain tissue was cut into small pieces, suspended in PBS and filtered through a 70 μm sieve. In order to obtain intestinal intraepithelial lymphocytes, the intestine was dissected from the stomach to the caecum and placed at a PBS drained tissue. At this stage, Peyer's patches and fatty tissue were removed, the intestine opened longitudinally, gently washed in PBS and cut into 1 cm pieces. The pieces were digested in 20 ml digestion medium (30 mM EDTA, 10% FCS in PBS) for 30 min at 37°C. To improve the suspension of intraepithelial lymphocytes, the tubes were shaken regularly. After 30 min, the tubes were vigorously shaken 10x and the suspension filtered through a tea sieve. The washing step was repeated twice by adding 10 ml digestion medium to intestine pieces followed by 10x shaking and filtration. IIEL were separated from the mucus using 37% percoll.

Cells were stained with the LIVE/DEAD Fixable Blue Dead Cell Stain Kit, for UV excitation (ThermoFisherScientific) and subsequently stained with antibodies for surface epitopes (CD3 AF488 (145-2C11), CD8 AF700 (53-6.7), CD19 PE (1D3), CD28 APC (37.51), CD44 PE-Cy7 (IM7), CD107a BV421 (1D4B), CD160 PerCP-Cy5.5 (7H1), PE-Cy7 (RMPI-30) from BioLegend, CD8 V450 (53-6.7), CD11a PE/V450 (M17/4), BTLA PE (6F7), γδ FITC (eBioGL3), HVEM APC (LH1), KLRG1 eF780 (2F1) eBioscience, CD4 V500 (RM4-5), PD-1 PE-Cy7 (PK136), TCRβ APC (H57-597) BD, CD45 FITC from Caltag). After fixation and permeabilisation (Foxp3/Transcription Factor Buffer Set from ThermoFisherScientific) intracellular staining was performed (GzmB AF647 (GB11), Ki67 AF488 (Ki-67) from BioLegend). Samples were recorded using the LSRII and analyzed using the FlowJo X 10.0.7r2 Treestar software. Gates were set according to fluorescence minus one (FMO) controls. Gating strategy is depicted in Supplementary Figure 4.

### Staining of mRNA for flow cytometry

In order to stain mRNA in addition to proteins for analysis by flow cytometry the PrimeFlow RNA Assay Kit from ThermoFisherScientific was used according to the manual provided. The probes included were directed against *Ifng, CD160*, and β*-Actin* (positive control) mRNA.

### PbA-specifc *in vitro* stimulation

Stimulation of PbA specific CD8^+^ T cells was performed with a mixture of three MHCI restricted PbA peptides [Pb1: SQLLNAKYL, Pb2: IITDFENL and F4: EIYIFTNI from Jerini Biotools (5)]. In order to pulse Hepa1-6 cells with peptides, complete RPMI (5% FCS, 1% L-Glutamine, 0.5% Gentamycine) was supplemented with 1 μg/ml of each peptide. After 3–4 h the medium was removed and cells were washed with PBS. Subsequently, CD8^+^ T cells were added in complete RPMI and co-cultured with the peptide-presenting cells. For detection of cytokines from cell culture supernatants, sandwich ELISAs (RnD) was performed according to the manual.

### Cytotoxicity assay

The cytotoxic effector function of CD8^+^ T cells was analyzed by a flow cytometry based assay (LIVE/DEAD cell mediated cytotoxicity Kit from ThermoFisherScientific) according to the manual. First, target cells, namely splenocytes from naïve mice, were pulsed with 1 μg/ml of Pb-peptides [Pb1: SQLLNAKYL, Pb2: IITDFENL and F4: EIYIFTNI from Jerini Biotools (5)] for 3 h at 37°C. Afterwards, cells were washed, diluted to 1x10^6^ cells/ml and stained with 0.25 μl/ml Dioctadecyloxacarbocyanine Perchlorate (DIOC) for 20 min in PBS. In order to remove DIOC from the supernatant, cells were washed twice in PBS and suspended in complete medium to a concentration of 1 × 10^6^ cells/ml. Second, effector cells from PbA infected mice at day 6 p.i. were sorted for CD44^hi^CD160^+/−^ cells and diluted to a concentration of 7.7 × 10^5^ cells/ml. To detect dead cells, a 1 μg/ml propidium iodide (PI) solution in complete medium was used. Samples containing 1 × 10^5^ effector cells, 1 × 10^4^ target cells and PI solution were cultured 2 h before analysis.

### Generation of CD160^−/−^ mice

CD160^−/−^ mice were generated using the CRISPR/Cas9 technology. The target region for gene editing was chosen using the online tool CHOPCHOP (36). The following oligos consisting of the T7 promotor and the target region were used: fw GAAATTAATACGACTCACTATAGGGAGAGCACAAGAAAGACGAAGCTGGTTTTAGAGCTAGAAATAGCAAGTTAAAATAAGGC; rev AAAAAAGCACCGACTCGGTGCCACTTTTTCAAGTTGATAACGGACTAGCCTTATTTTAACTTGCTATTTCTAGCTCT. Oligos were amplified by PCR, purified by agarose gel electrophoresis and extracted with the Qiaex II Gel Extraction Kit (Quiagen) according to the manual. The purified PCR products were used as templates for transcription into RNA. sgRNA and Cas9 protein were injected into cytoplasm and pronucleus of one-cell staged C57BL/6 mouse embryos and implanted into foster mothers. The heterozygous offspring was used as founder animals and crossed with C57BL/6J mice. Two knockout lines derived from different founder animals were bred and characterized. Because both lines behaved alike, only one was chosen for further experiments. Genotyping of mice was performed by restriction-length fragment polymorphism (RLFP). Tissue samples were lysed and genomic DNA sequences consisting of the target region were amplified by PCR using the lysate as a template. Subsequently, PCR products were digested by an enzyme specific for the wild type sequence. Hence, wild type DNA is digested into two smaller fragments, while modified DNA remains at the original size.

### Statistical analysis

Statistical analysis was performed using Graph Pad Prism 5.0b. The statistical significance between the two groups was calculated using the two-tailed Mann-Whitney test while correlation was calculated using the Spearmann Correlation test. ^*^p < 0.05, ^**^p < 0.01, and ^***^p < 0.001.

## Results

### HVEM controls CD8^+^ T cell persistence during malaria blood-stage

**Figure 1:**
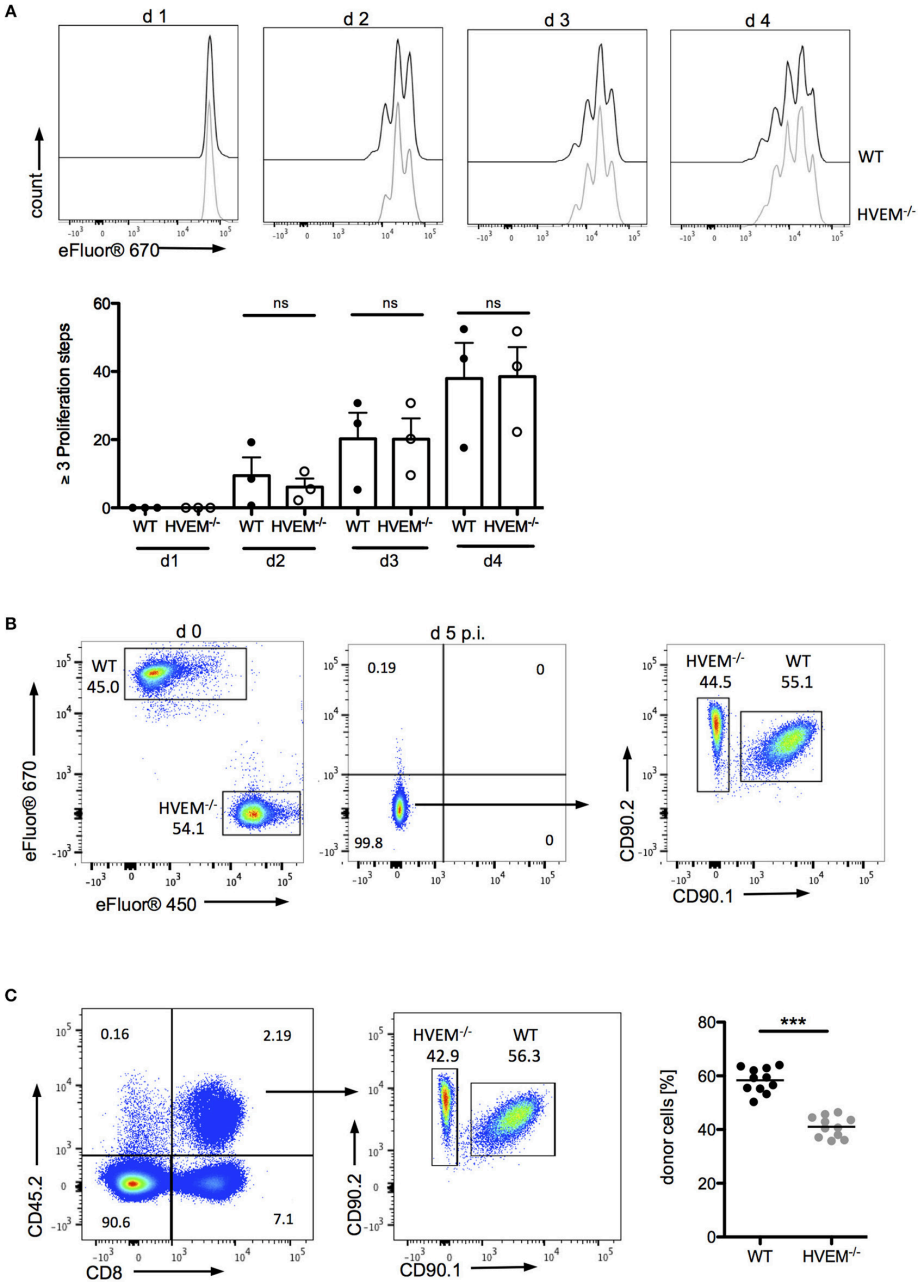
HVEM is required for CD8^+^ T cells persistence during malaria blood-stage. **(A)** Splenocytes of WT OT-1 or HVEM^−/−^ OT-1 mice were labeled with the proliferation dye eFluor670 and stimulated for 1–4 days with the MHCI-restricted peptide SIINFEKL. Representative histograms are shown. Biological replicates from one experiment, which is representative of two independent experiments, are reported. The means ± SEM are depicted, each dot represents one mouse. For adoptive transfer assays, WT OT-1 and HVEM^−/−^ OT-1 CD8^+^ T cells were labeled with eFluor670 (WT) or eFluor450 (HVEM^−/−^) proliferation dyes, respectively and transferred in a 1:1 ratio into a C57BL/6 host, which was concomitantly infected with Pb-OVA. At d 5 p.i. proliferation and persistence of transferred CD8^+^ T cells was analyzed by **(B)** dilution of fluorescent dyes and **(C)** frequency of cells expressing the congenic markers CD45.2/CD90.2. Representative plots are depicted. Data were pooled from three independent experiments performed with 2–5 mice/group (total *n* = 11). ^***^p < 0.0001.

The role of co-stimulatory signaling by HVEM on proliferation of CD8^+^ T cells was first evaluated using antigen-specific *in vitro* stimulation of wild type (WT) OT-1 and HVEM^−/−^ OT-1 CD8^+^ T cells. In this experimental setting, no differences in the proliferation of WT and HVEM^−/−^ OT-1 CD8^+^ T cells could be observed (Figure 1A). Next, we analyzed not only the proliferative capacity, but also the persistence of CD8^+^ T cells during an acute *P. berghei* ANKA (PbA) infection by a competitive, adoptive T cell transfer assay in which WT OT-1 and HVEM^−/−^ OT-1 CD8^+^ T cells, labeled with different proliferation dyes, were mixed in equal proportions and transferred into WT hosts. Concomitantly, mice were infected with *P. berghei*-OVA (Pb-OVA). In this experimental setting, both CD8^+^ T cell populations that can be tracked according to their different congenic markers (both T cell populations: CD45.2^+^; WT OT-1: CD90.1^+^; HVEM^−/−^ OT-1: CD90.1^−^; recipient: CD45.1^+^CD45.2^−^) are exposed to the same inflammatory environment and parasitemia level, thus specifically allowing the analysis of the HVEM function. Of note, both WT OT-1 and HVEM^−/−^ OT-1 T cells were able to completely dilute the proliferation dye by day 5 post infection (p.i.) (Figure 1B). However, WT CD8^+^ T cells persisted longer during Pb-OVA infection after adoptive T cell transfer compared to the HVEM^−/−^ CD8^+^ T cell counterpart (Figure 1C). Taken together, co-stimulation by HVEM is not required for initial expansion of effector cells, but crucial for their maintenance even during the first 5 days of blood-stage malaria.

### Cerebral malaria development during PbA infection is dependent on HVEM engagement on CD8^+^ T cells

**Figure 2:**
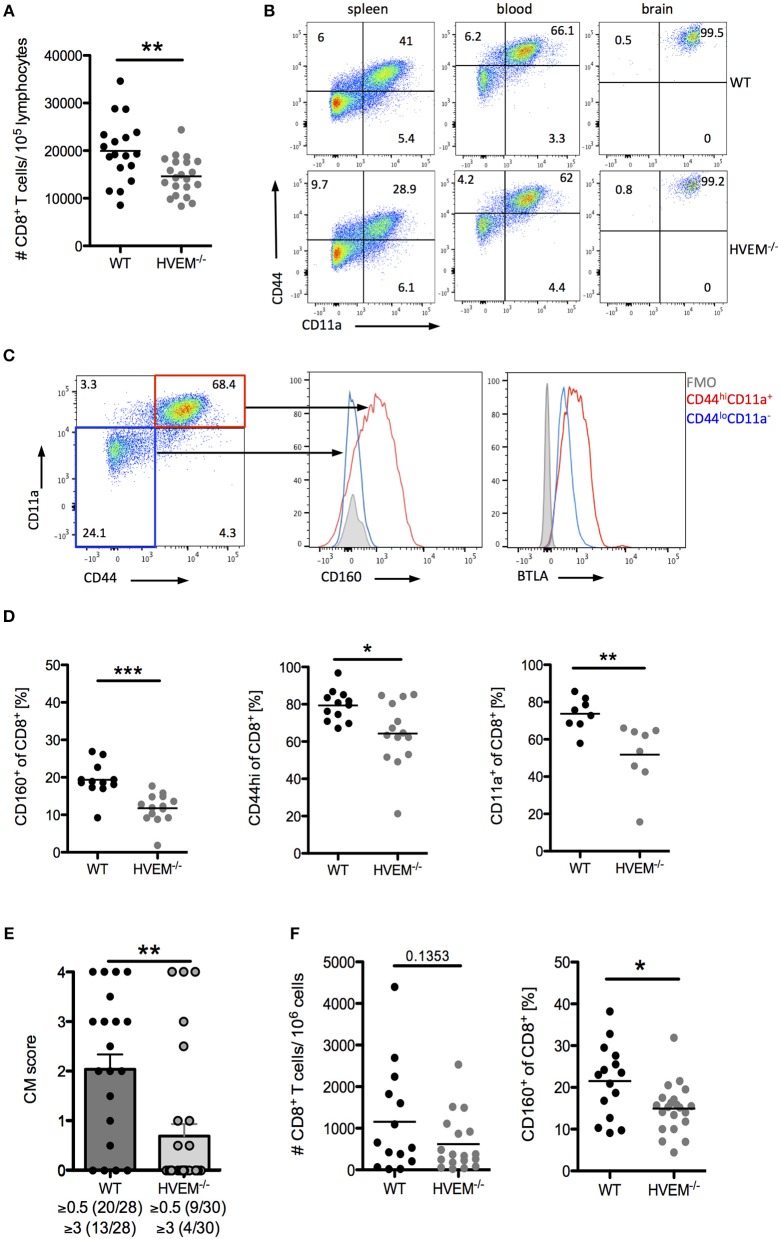
HVEM engagement augments the development of cerebral malaria. Flow cytometric analysis of CD8^+^ T cells in WT and HVEM^−/−^ mice at d 6 post PbA infection. **(A)** Number of CD8^+^ T cells in the blood. Pooled data of 5 independent experiments performed with 3–5 mice/group (WT *n* = 18, HVEM^−/−^ *n* = 21). **(B)** Representative dot plots of CD44 and CD11a staining of CD8^+^ T cells derived from spleen, blood or brain of wild type or HVEM^−/−^ mice. **(C)** Expression of CD160 or BTLA within the CD44^lo^CD11a^−^ (blue) or CD44^hi^CD11a^+^ (red) cell population. **(D)** Frequency of CD160^+^, CD44^hi^ or CD11a^+^ cells in the blood gated on CD8^+^ T cells. Data were pooled from 2 (CD11a) or 3 (CD44, CD160) independent experiments performed with 3–6 mice/group (CD11a: WT *n* = 8, HVEM^−/−^ *n* = 8; CD44: WT *n* = 12, HVEM^−/−^ *n* = 14; CD160: WT *n* = 12, HVEM^−/−^ *n* = 14). ^*^p < 0.05, ^**^p < 0.01, and ^***^p < 0.001. **(E)** Severity of cerebral symptoms was monitored in WT and HVEM^−/−^ mice at d 6 p.i. Data were pooled from 8 independent experiments including 3–6 mice/group. ^**^p = 0.0014. **(F)** Number of brain-infiltrating CD8^+^ T cells and frequency of CD160^+^ T cells within the CD8^+^ population. Data were pooled from 4–5 independent experiments including 2–5 mice/group (#CD8: WT *n* = 14, HVEM^−/−^ *n* = 18; CD160: WT *n* = 15, HVEM^−/−^ *n* = 21). ^*^p = 0.0107.

Infection of C57BL/6 mice with PbA causes disruption of the blood-brain barrier by cytotoxic CD8^+^ T cells (5). When we examined the CD8^+^ T cell pool in WT and HVEM^−/−^ mice infected with the parasite, in line with our adoptive transfer experiment results (Figure 1C), we found significant reduction in CD8^+^ T cell numbers in the blood of HVEM^−/−^ mice compared to the control counterpart (Figure 2A). However, in contrast to OT-1 cells, which were used for the adoptive T cell transfer experiment, the pool of CD8^+^ T cells in WT and HVEM^−/−^ mice represents PbA specific and unspecific, naïve CD8^+^ T cells. In order to analyze CD8^+^ T effector cells, we examined the expression of the activation marker CD44 and the marker for antigen-experienced cells CD11a (37–39). In contrast to spleen and blood, where only a fraction of CD8^+^ T cells co-expressed these markers, all brain-infiltrating cells were CD44^hi^ and CD11a^+^. Thus, we considered this marker combination suitable for the detection of effector cells (Figure 2B). We have previously shown that the co-inhibitory receptor and HVEM-ligand BTLA is expressed on both activated and naïve CD8^+^ T cells (7). In contrast to BTLA, we found that CD160, another HVEM-ligand sharing the binding region with BTLA, is selectively expressed on CD8^+^CD44^hi^CD11a^+^ T cells, but not on naïve T cells (Figure 2C). Surprisingly, we observed a reduced frequency of CD8^+^CD160^+^ T cells in the blood of PbA infected HVEM^−/−^ mice compared to the WT counterpart, and a diminished percentage of activated (CD8^+^CD44^hi^) and antigen-experienced (CD8^+^CD11a^+^) CD8^+^ T cells (Figure 2D). Considering the detrimental role of CD8^+^ T cells in the development of cerebral malaria, we thus monitored WT and HVEM^−/−^ mice for the progression of cerebral symptoms. Indeed, PbA infected HVEM^−/−^ mice developed less severe symptoms in terms of neurological defects compared to WT mice (Figure 2E). Interestingly, even though the total number of CD8^+^ T cells in the brain was unchanged (Figure 2F), the frequency of CD8^+^CD160^+^ T cells was substantially reduced in HVEM^−/−^ mice (Figure 2F) compared to WT controls. These findings show that HVEM is essential for regulating the magnitude of CD8^+^ T effector cell activation, mirrored by the reduced expression of CD160, thereby controlling the development of cerebral malaria.

### CD160 characterizes highly activated and cytotoxic CD8^+^ T cells

**Figure 3:**
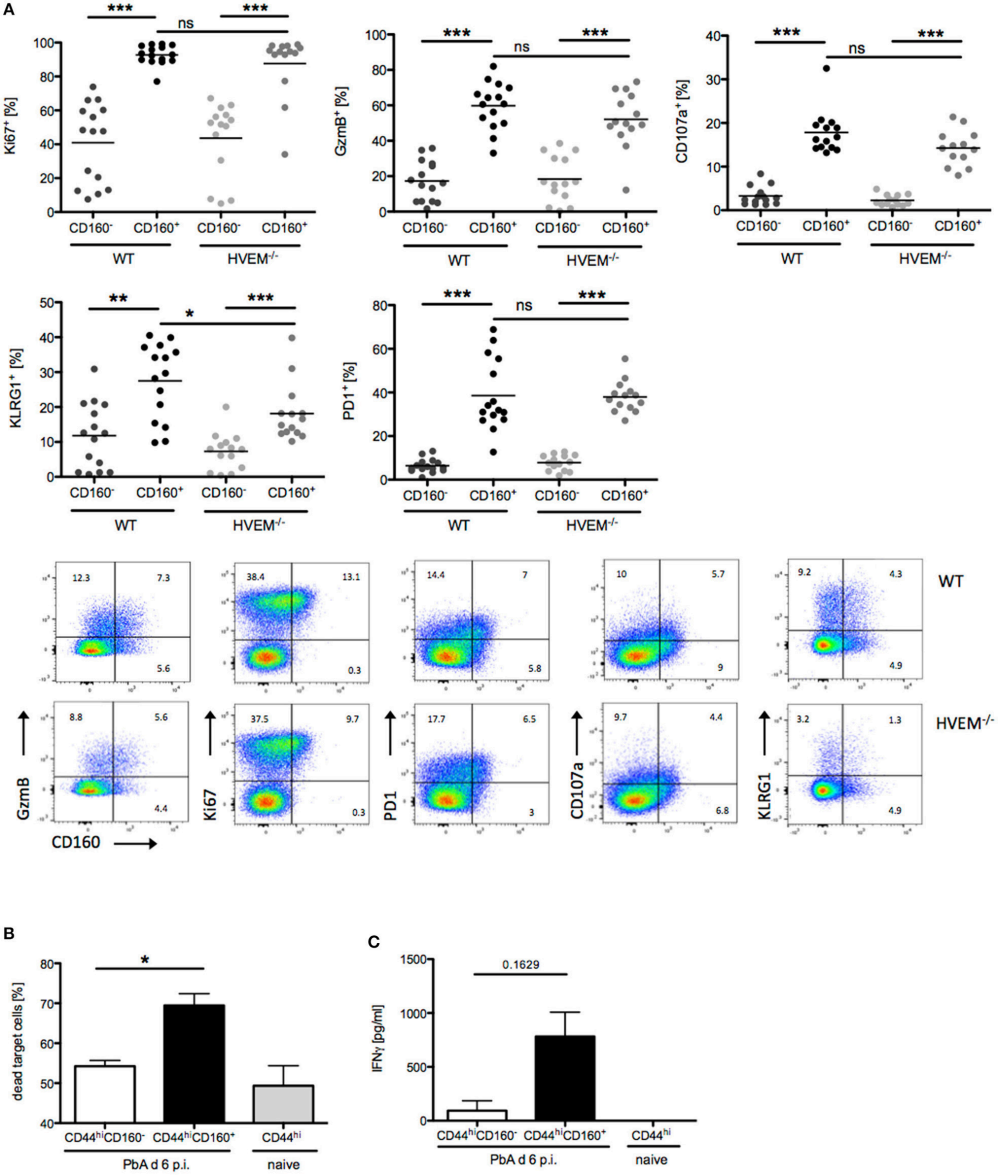
CD160 characterizes highly activated and cytotoxic CD8^+^ T cells during malaria. **(A)** Flow cytometric analysis of CD160^+/−^CD8^+^ T cells isolated from the spleen at d 6 p.i. Data were pooled from 3 independent experiments including 3–6 mice/group (total WT *n* = 14–15, HVEM^−/−^ *n* = 13–14). Representative plots are shown. ^*^p < 0.05, ^**^p < 0.01, and ^***^p < 0.001. **(B)** Flow cytometry based cytotoxicity assay performed on CD8^+^CD44^hi^CD160^+^ and CD8^+^CD44^hi^CD160^−^ effector cells FACS sorted from spleens of PbA infected mice at d 6 p.i., and co-cultured with naive splenocytes pulsed with Pb-specific peptides as target cells. Data were pooled from 4 independent experiments, in which a pool of splenocytes from 3–4 mice was used. This means ± SEM are shown. ^*^p = 0.0286 **(C)** Release of IFNγ by effector cells, isolated as described in **(B)**, after 24 h co-culture with Pb-peptide pulsed Hepa1-6H cells. Pooled data of three independent experiments performed in at least triplicates is shown as means ± SEM.

In light of our results on the selective expression of CD160 on CD8^+^CD44^hi^CD11a^+^ T cells, their strong reduction in frequency and concurrent reduction of pathology in HVEM^−/−^ mice, we further characterized the signature profile of CD8^+^CD160^+^ T cells. First, we observed that CD8^+^CD160^+^ T cells were enriched for markers of proliferation (Ki67), cytotoxicity (GzmB), degranulation (CD107a), and differentiation (KLRG1, PD-1) compared to CD8^+^CD160^−^ T cells, independently of whether they were derived from WT or HVEM^−/−^ mice (Figure 3A). Second, expression of KLRG1 was lower in CD8^+^CD160^+^ T cells of HVEM^−/−^ mice than in WT control. These data, together with the fact that a reduced frequency of CD8^+^CD160^+^ T cells have been observed in HVEM^−/−^ mice (Figures 2D,F) and support the hypothesis that HVEM signaling is required for persistence of a population of terminally differentiated CD8^+^ T cells during PbA infection.

In order to provide evidence of the actual functionality of CD8^+^CD160^+^ T cells, we devised *in vitro* assays in which CD8^+^CD44^hi^CD160^+^ T cells from PbA infected mice (d 6 p.i.) were FACS sorted and analyzed for their ability to kill target cells and to produce the pro-inflammatory cytokine IFNγ. CD8^+^CD44^hi^CD160^−^ T cells from PbA infected mice and CD8^+^CD44^hi^ T cells from naïve mice were used as internal control. In line with the difference in the cytotoxic characteristic observed (Figure 3A), CD160^+^ cells were more effective in killing target cells, compared to the similarly activated (CD44^hi^) CD160^−^ counterpart (Figure 3B). Additionally, CD160^+^ cells showed a trend to produce higher amounts of IFNγ compared to the control cells. However, no statistical significance was reached, which might be due to inter experimental variations (Figure 3C). Taken together, these data identify CD160 as signature of CD8^+^ T cell with high killing capacity and IFNγ production in the context of blood-stage malaria.

### CD8^+^CD160^+^ T cells are associated with cerebral pathology

**Figure 4:**
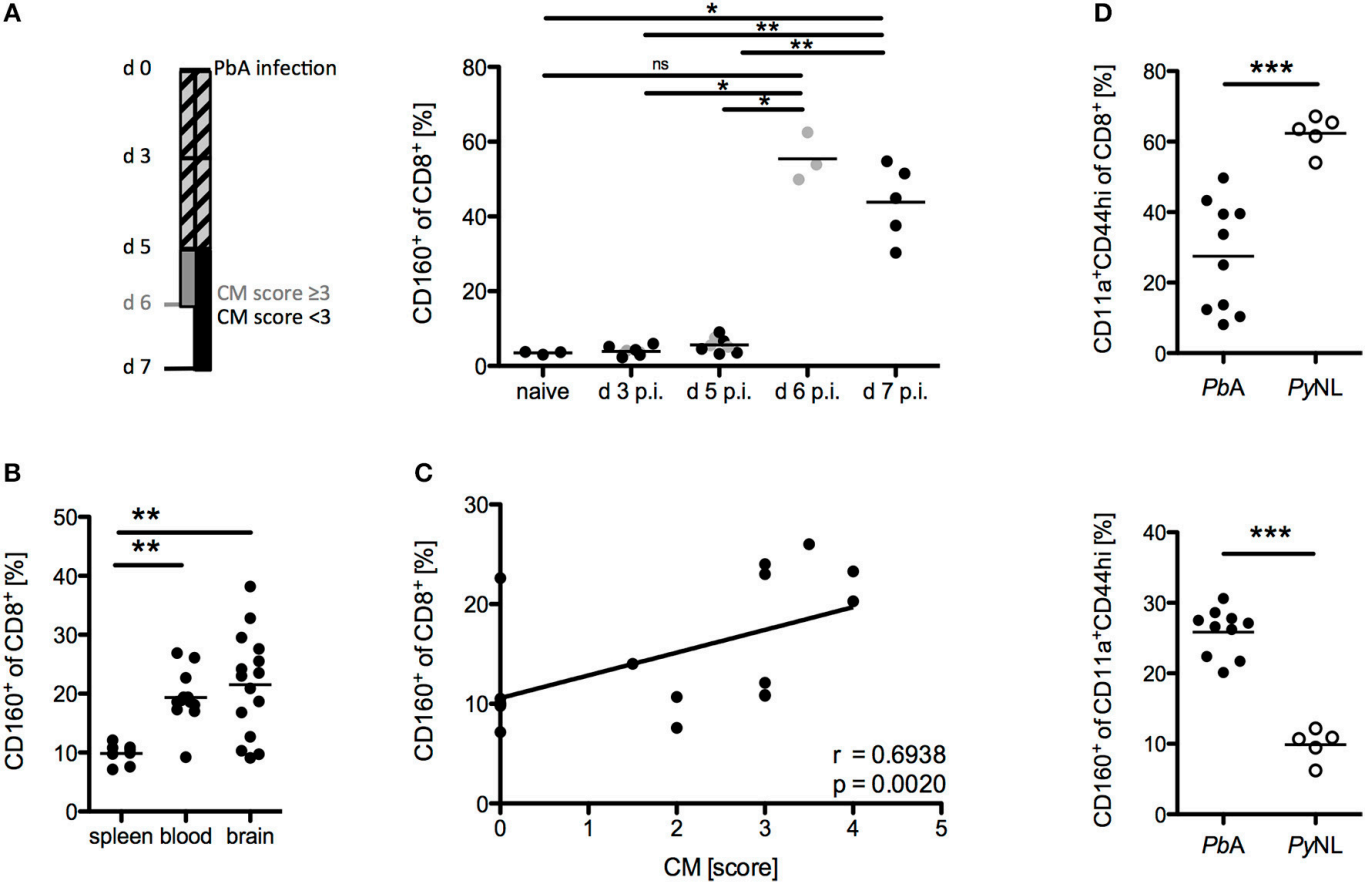
CD8^+^CD160^+^ T cells are associated with cerebral pathology during PbA infection. **(A)** Flow cytometric analysis of the frequency of CD8^+^CD160^+^ T cells in the blood of WT mice, collected at the indicated days post PbA infection. Mice that developed severe cerebral malaria by d 6 p.i. (indicated in gray) were analyzed at day 6 whereas all other mice (indicated in black) were analyzed at d 7 p.i. Data were pooled from two independent experiments including up to 5 mice/group (naive *n* = 3, PbA *n* = 8) **(B)** Flow cytometric analysis of the frequency of CD8^+^CD160^+^ T cells in spleen, blood or brain of PbA infected WT mice. Data was pooled from 6 independent experiments including 2–4 mice/group (spleen *n* = 8, blood *n* = 12, brain *n* = 15) **(C)** Correlation of the severity of cerebral symptoms and frequency of splenic CD8^+^CD160^+^ T cells of PbA infected WT mice. Data were pooled from 4 independent experiments including 4–5 mice/group (total *n* = 17). **(D)** Frequency of activated (CD11a^+^CD44^hi^) CD8^+^ T cells and CD160^+^ T cells within the CD11a^+^CD44^hi^ cell population isolated from spleens of either PbA or PyNL infected mice. Data were pooled from two independent experiments including 2–5 mice/group (PbA *n* = 10, PyNL *n* = 5). ^*^p < 0.05, ^**^p < 0.01, and ^***^p < 0.001.

The phenotype and functional properties of CD8^+^CD160^+^ T cells suggest their involvement in cerebral pathology. Hence, we further correlated CD160 expression on CD8^+^ T cells with the development of cerebral malaria. Using our protocol, severe cerebral symptoms in the PbA infection model occurred at day 6 p.i. with most of the mice succumbing to cerebral pathology between day 6 and 8 p.i. CD160 expression on CD8^+^ T cells was thus analyzed early after infection (d 3 p.i,), before first symptoms occurred (d 5 p.i.), upon onset of severe symptoms (d 6 p.i.) and at the peak of T cell activation (d 7 p.i.). Strikingly, CD160 remained absent until d 5 p.i. and was clearly induced at the peak of pathology (days 6–7 p.i.) (Figure 4A). The highest frequency of CD8^+^CD160^+^ T cells was detected in blood and brain where the most tissue damage occurs, compared to the spleen (Figure 4B) where the parasitized RBCs are eradicated and T cells are activated (40). Importantly, the frequency of CD8^+^CD160^+^ T cells correlated with the severity of cerebral symptoms (Figure 4C).

Furthermore, we examined the induction of CD160 on CD8^+^ T cells in a non-lethal model of malaria via injection of the parasite *Plasmodium yoelii* (PyNL). PyNL causes strong T cell activation (CD44^hi^CD11a^+^) compared to the lethal parasite strain (PbA). However, we observed reduced frequency of CD8^+^CD160^+^ T cells within the activated CD8^+^ T cell population in non-lethally (PyNL) vs. lethally infected (PbA) WT mice (Figure 4D). Because mice are able to naturally clear PyNL parasite, we also analyzed CD160 expression on CD8^+^ T cells after resolution of the infection (d 20 p.i.). Interestingly, CD8^+^ T cells maintained stable CD160 expression up to day 20 p.i., even though their proliferation capacity is drastically reduced as shown by the down-regulation of the Ki67 epitope (Supplementary Figure 1).

Taken together, there are several findings that strongly suggest that CD160 contributes to CD8^+^ T cell regulation in particular, in highly activated CD8^+^ T cells, which are harmful in PbA infection: The kinetics of CD160 expression; tissue distribution of the CD160^+^ cells; correlation of the frequency of CD160^+^ cells and cerebral symptoms; and their reduction in the non-lethal plasmodial infection.

### CD160 restricts T cell mediated immunopathology

**Figure 5:**
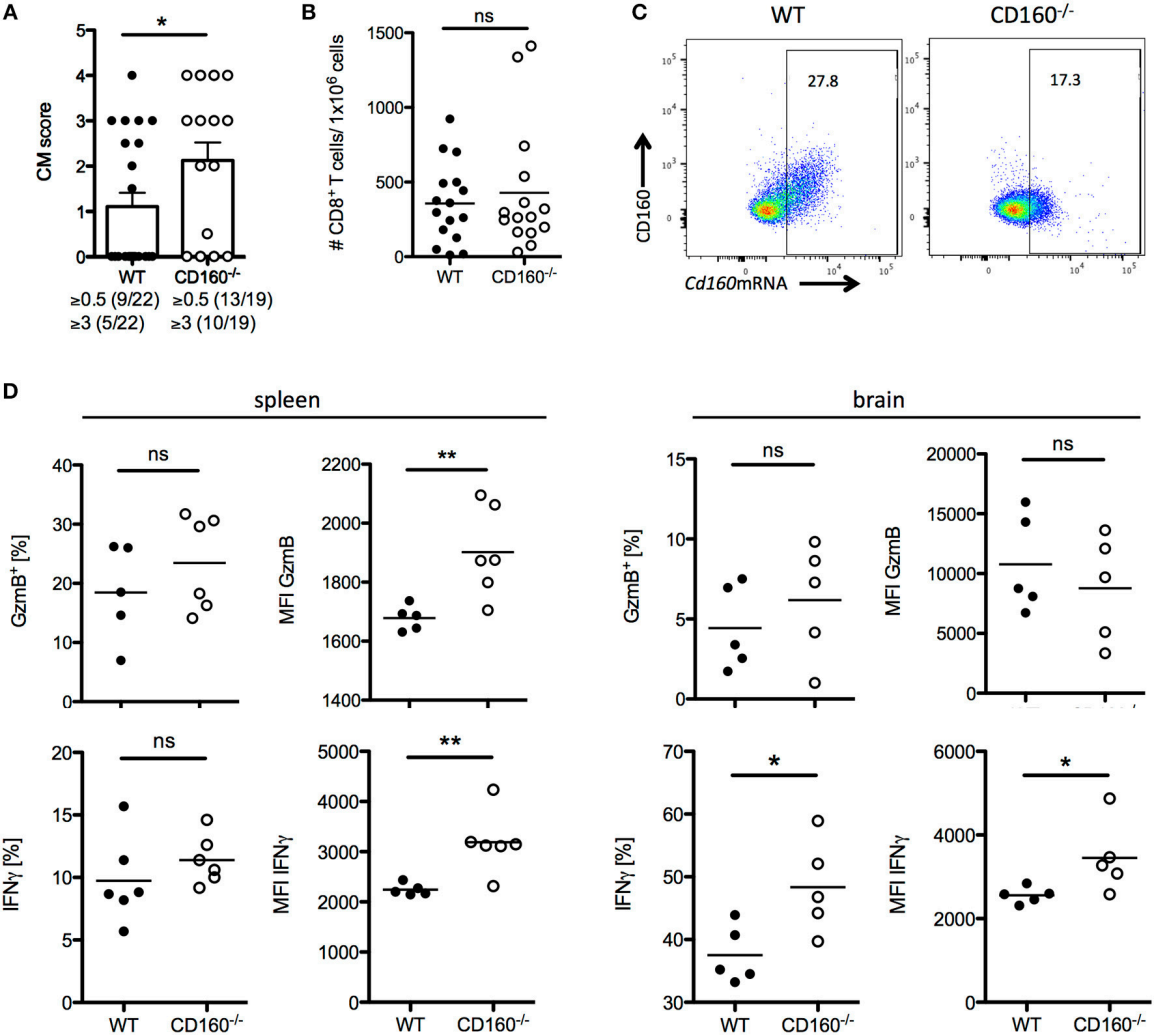
CD160 restricts T cell mediated cerebral pathology. **(A)** Severity of cerebral symptoms was monitored in WT and CD160^−/−^ mice at d 6 post PbA infection. Data were pooled from 4 independent experiments including 3–9 mice/group. **(B)** Absolute number of brain-infiltrating CD8^+^ T cells in PbA infected WT and CD160^−/−^ mice was measured by flow cytometry. Data were pooled from 3 independent experiments including 3–8 mice/group (WT *n* = 16, CD160^−/−^ *n* = 15). **(C)** Representative dot plots of CD8^+^ T cells from the spleen from PbA infected mice stained for CD160 protein and *Cd160* mRNA. **(D)** CD8^+^*Cd160* mRNA^+^ T cells from WT or CD160^−/−^ mice were analyzed for frequency (%) and mean fluorescence intensity (MFI) of granzyme B (protein) and IFNγ (mRNA). Representative data from one out of two experiments is shown. ^*^p < 0.05 and ^**^p < 0.01.

A co-stimulatory role for CD160 has been previously suggested in NK cells and T cells in the context of allograft rejection, melanoma and lymphoma tumor models (41–43). Importantly, our data during malaria now strongly support the idea of CD160 as a critical regulator of CD8^+^ T cytotoxicity and consequent detrimental function on the overall outcome of malaria. However, it seems reasonable that co-inhibitory receptors are induced especially on highly active cells in order to restrict T cell function and CD160 has already been described as a co-inhibitory receptor on human CD4^+^ T cells (29). To address the role of CD160 in CD8^+^ T cells in the PbA model, we generated CD160^−/−^ mice using the CRISPR/Cas9 technology on C57BL/6 background (Supplementary Figure 2). Because CD8^+^ T cells are the key players in the induction of cerebral malaria, we first evaluated the severity of symptoms at day 6 p.i. CD160^−/−^ mice developed more severe cerebral malaria compared to WT mice (Figure 5A), although they exhibit a similar parasitemia (Supplementary Figure 3). Considering the similar number of brain-infiltrating CD8^+^ T cells observed (Figure 5B), we further investigated their phenotypical profile. Only a fraction of CD8^+^ T cells expressed CD160 in WT mice. However, due to the lack of a surrogate marker for CD8^+^CD160^+^ T cells, the isolation of this cell population in CD160^−/−^ mice was not possible. Importantly, in the CD160^−/−^ mice a deletion of 5 amino acids was introduced resulting in a premature stop codon (Supplementary Figure 2). However, the *Cd160* mRNA upstream of the deletion site is unchanged compared to the WT sequence. Therefore, we could detect *Cd160* mRNA both in WT and CD160^−/−^ cells using a probe binding upstream of the deletion site (Figure 5C). We used *Cd160* mRNA probes for selective identification of *Cd160* mRNA^+^ CD8 T cells in WT and CD160^−/−^ mice. Although no differences in the frequency of GzmB or IFNγ expressing cells within the splenic CD8^+^*Cd160*mRNA^+^ T cells was observed, increased mean fluorescence intensity (MFI) for both molecules was detected in CD160^−/−^ CD8^+^ T cells compared to controls, thus confirming that CD160 controls CD8^+^ T cell cytotoxic function and IFNγ production. Of note, while CD160 did not regulate GzmB secretion, IFNγ production was still a CD160-dependent process in CD8^+^ T cells in the brain (Figure 5D).

### CD8^+^CD160^+^ T cells exhibit a similar phenotype in human and mice

**Figure 6:**
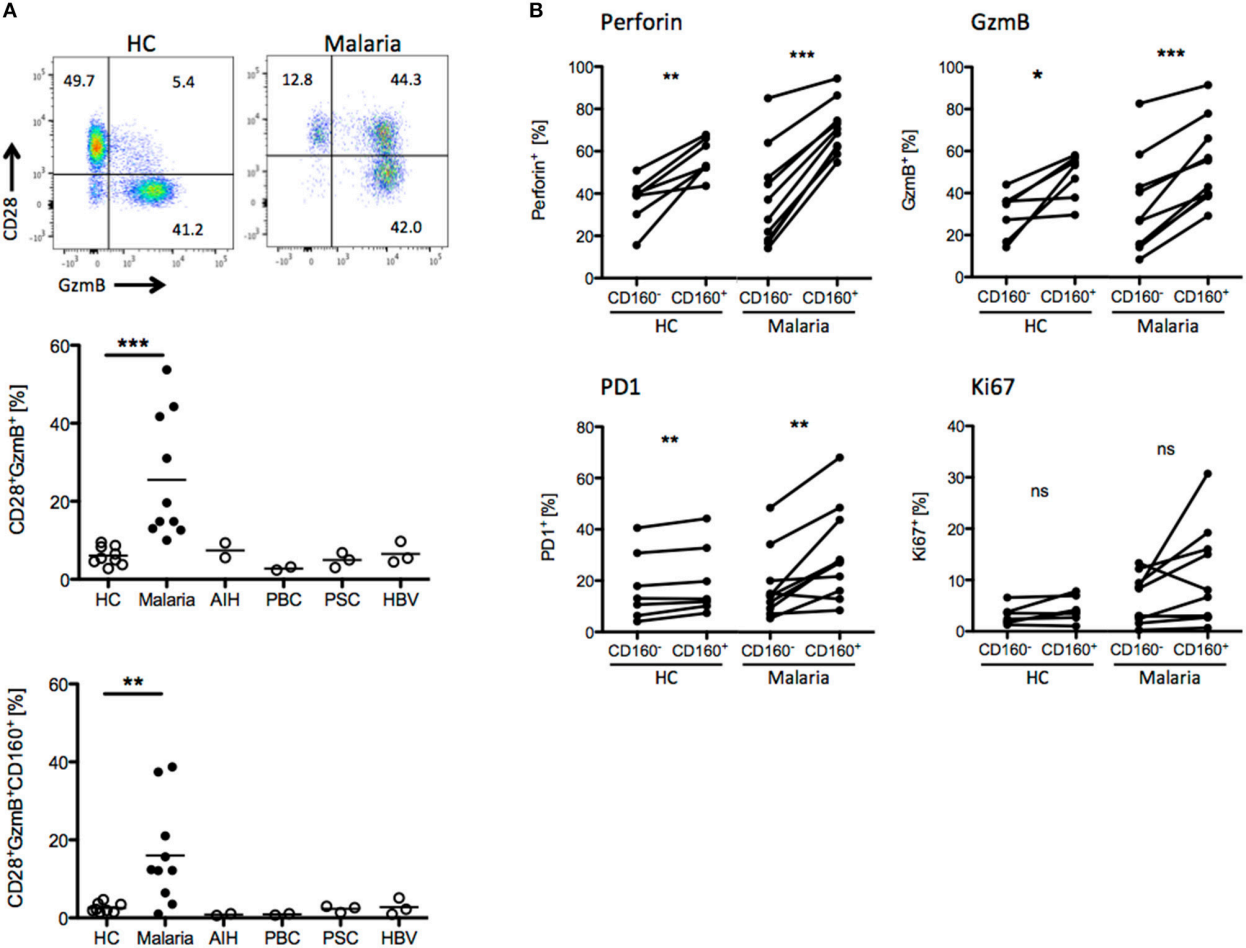
CD8^+^CD160^+^ T cells exhibit a similar phenotype in human and mice. **(A)** Whole peripheral blood samples from healthy controls (HC, *n* = 9), Malaria patients (Malaria, *n* = 10) and patients suffering from autoimmune hepatitis (AIH, *n* = 2), primary biliary cholangitis (PBC, *n* = 2), primary sclerosing cholangitis (PSC, *n* = 3) and chronic hepatitis B virus infection (HBV, *n* = 3) were analyzed by flow cytometry for the expression of CD28, GzmB, and CD160 on CD8^+^ T cells. Representative plots are shown. **(B)** Expression of Perforin, GzmB, PD1, and Ki67 in CD8^+^CD160^+^ and CD8^+^CD160^−^ T cells isolated from healthy controls (*n* = 7) or malaria patients (*n* = 10) was analyzed by flow cytometry. ^*^p < 0.05, ^**^p < 0.01, and ^***^p < 0.001.

We compared the T cell phenotypes in human peripheral blood of malaria patients and healthy controls (HC) in light of the previous findings in the mouse model. Unfortunately, tools for the staining of *Plasmodium*-specific CD8^+^ T cells have not been developed yet. Additionally, high background level of CD8^+^ T cells activated by e.g., previous or chronic viral infection might interfere with recent *Plasmodium*-specific CD8^+^ T cell activation. For this reason, we investigated the presence of CD8^+^ T cell biomarkers specifically induced during malaria. As additional controls, we used peripheral blood samples from patients suffering from autoimmune liver disease [autoimmune hepatitis (AIH), primary billary cholangitis (PBC), primary sclerosing cholangitis (PSC)] or chronic hepatitis B virus (HBV) infection. CD8^+^ T cells were stained for activation (CD28), proliferation (Ki67), cytotoxicity (GzmB, Perforin) and differentiation markers (PD-1). We found that CD8^+^CD28^+^GzmB^+^ T cells were enriched in malaria patients compared to healthy controls and patients suffering from AIH, PBC, PSC or chronic HBV infection (Figure 6A). Indeed, CD160 was co-expressed with GzmB and CD28, suggesting that it is induced during acute *Plasmodium* infection in CD8^+^ T cells (Figure 6A). Subsequently, we analyzed the expression of the cytotoxic molecule GzmB and Perforin the proliferation marker Ki67 and the co-inhibitory receptor PD-1, both in CD8^+^CD160^+^ and CD8^+^CD160^−^ T cells from healthy controls or malaria patients. While no difference in Ki-67 expression was observed, the frequency of cytotoxic (GzmB^+^ and Perforin^+^) and terminally differentiated (PD-1^+^) cells was increased in CD8^+^CD160^+^ T cells compared to the control counterpart (Figure 6B).

Taken together, our results show that CD160 expression identifies a population of highly activated and cytotoxic CD8^+^ T cells in peripheral blood. Of note, this feature is conserved in murine and human CD8^+^ T cells.

## Discussion

A tight control of CD8^+^ T effector cell function is crucial in order to allow efficient clearance of invading pathogens without the development of immunopathology due to an overwhelming response. Considering that co-inhibitory and co-stimulatory receptors are important regulators of CD8^+^ T effector function, we aimed to better understand how the HVEM-CD160 axis shapes the CD8^+^ T cell response during malaria.

HVEM is broadly expressed in CD4^+^ and CD8^+^ T cells (15). In this study we focused on CD8^+^ T cells since they are central for mediating cerebral pathology. By taking advantage of adoptive transfer experiments of control and HVEM-deficient CD8^+^ T cells in the same WT recipient, we can address the intrinsic and direct contribution of HVEM on CD8^+^ T cell function. In view of the equal proliferation observed in CD8^+^ T cells from WT and HVEM^−/−^ mice in *in vivo* and *in vitro* experimental settings, we conclude that HVEM seems to provide pro-survival signals, which are essential for the persistence of CD8^+^ T effector cells. Consequently, the number and frequency of terminally differentiated effector cells is reduced. However, CD8^+^ T cells lacking HVEM are not impaired in their capacity to express e.g., the effector molecule GzmB. Our data on the role of HVEM in the control of CD8^+^ T cell persistence are supported by studies showing the pro-survival function of HVEM in memory T cells performed in the context of listeria, influenza and vaccinia virus infection or with the model antigen OVA (19–22). Our data suggest that the reduced frequency of highly activated CD8^+^ T cells, which can be identified by the expression of CD160, results in the reduced severity of ECM.

The HVEM-ligand CD160 has been reported to be expressed by cytotoxic and IFNγ producing NK and CD8^+^ T cells (31, 32, 44). Based on this, we analyzed its function in CD8^+^ T cells during ECM. CD160 competes with the co-inhibitory receptor BTLA for the binding to HVEM. We have previously shown that targeting BTLA with an agonistic antibody can restrict cerebral pathology (7). However, BTLA is expressed by a variety of cell types including innate and adaptive immune cells. Consequently, BTLA not only restricts the T cell mediated immunopathology during cerebral malaria but also the protective immunity against a non-lethal infection with *P. yoelii* by the suppression of phagocytes and B cells (8). In contrast to BTLA, our data shows that CD160 expression is restricted to highly activated CD8^+^ T cells.

The cytotoxic phenotype of CD8^+^ T cells, which express CD160 has already been described by others (44). Only one study has indeed analyzed the direct function of CD160 in NK cells by using a CD160^−/−^ mouse model. In this scenario, CD160 deficient NK cells showed in melanoma and RMA-S lymphoma tumor models a defect in tumor control due to impaired IFNγ production (41). Our results regarding CD8^+^ T cells hint into a different direction. First, CD160^−/−^ mice develop more severe cerebral pathology suggesting a co-inhibitory rather than a co-stimulatory function of CD160. Second, we observed an increase in IFNγ mRNA in CD8^+^ T cells of CD160^−/−^ mice. The role of CD160 might be different in NK and CD8^+^ T cells because of a different composition of co-receptors expressed by the respective cell types. In T cells, engagement of CD160 leads to association to the CD3ζ chain (29, 30). In contrast, in NK cells CD160 has been described to co-localize with CD2 (45). Furthermore, the CD160-ligand might also influence the effect of CD160 engagement. Besides HVEM, CD160 binds to classical and non-classical MHCI molecules (27, 31, 32, 46). A dual function of co-receptors depending on e.g. the ligand or the cell type they are expressed by has already been described for other receptors such as 2B4 and Tim-3 (47, 48).

It might be possible that the lack of CD160 disturbs the balanced signaling between co-stimulatory HVEM and co-inhibitory BTLA. However, this is unlikely due to the high expression levels of BTLA and HVEM compared to the low level of CD160 during ECM. But even if HVEM signaling is impaired by the loss of CD160, this should lead to reduced immunopathology according to our data obtained in HVEM^−/−^ mice. However, we see an aggravated pathology in CD160^−/−^ mice. Hence we conclude, that the phenotype is directly mediated by the loss of CD160 rather than by influencing the HVEM-BTLA axis.

In order to analyse the function of CD160, besides its genetic ablation, several studies utilized soluble CD160 molecules or antibodies to disrupt the interaction of CD160 with its respective ligand in different experimental settings. In a model of cardiac allograft transplantation, treatment with CD160Ig was beneficial to control CD8^+^ mediated allograft rejection (42). The authors propose that in this context CD160 acts as a co-stimulatory molecule on CD8^+^ T cells and thus the use of CD160Ig reduces the amount of secreted IFNγ by CD8^+^ T cells (42). Similarly, CD160Ig treatment enhances the graft survival in a model of skin allograft rejection when treatment is combined with anti-CD40L antibody (43). Of note, when treated with soluble CD160 not only is the CD160-HVEM interaction affected due to their shared binding region, but so is the binding of BTLA to HVEM. Furthermore, it is not clear whether the soluble CD160 preferentially binds to either HVEM or MHCI or both but the higher binding affinity of CD160 for HVEM suggest that it binds to HVEM. In this case, the signaling of CD160 expressed by CD8^+^ T cells might not be completely blocked but instead interaction with MHCI is favored. Additionally, in mice infected with *Citrobacter rodentium*, CD160 expressed by intraepithelial lymphocytes triggers HVEM on epithelial cells and induces an anti-microbial response in conjunction with IL-22 (28). Finally, *in vitro* cross-linking of CD160 is reported to either stimulate or inhibit the expressing cells, depending on co-stimulation and cell type (15, 27, 29, 30, 31, 49, 50, 51). In summary, most studies suggest a co-stimulatory role of CD160. However, it is important to consider that the antibody could have an agonistic rather than a blocking function. Because of these limitations we decided to generate a CD160-deficient mouse.

In contrast to their absence in naïve mice, we detect CD8^+^CD160^+^ T cells in peripheral blood of healthy donors. Other groups have already described CD160 expression in CMV, EBV and HIV-specific CD8^+^ T cells in chronically infected patients (49, 52, 53). These data are in line with our results, which show a constant expression of CD160 in CD8^+^ T cells from mice infected with PyNL even after natural clearance of the parasite. Taken together, these data suggest that once CD160 is induced on murine and human CD8^+^ T cells, its expression is maintained and that this expression is associated with increased levels of CD8^+^ T cell activation and cytotoxicity.

CD160 has been selected as a candidate target for the treatment of vascular-eye diseases. Furthermore, it has also been considered for therapeutic intervention in cancer patients, according to the anti-angiogenic effect of CD160 antibodies on growing endothelial cells (54, 55). Based on our data, we propose that CD160 delineates highly activated CD8^+^ T cells and might be useful to restrict immunopathology in the future.

In conclusion, in this study we have shown that the HVEM-CD160 axis is critical in the fine-tune regulation of stimulatory and inhibitory signals in CD8^+^ T cells during blood-stage malaria.

## Author contributions

FM and TJ designed the experiments. FM and NS conducted the experiments. FM, NS, TJ, and LB analyzed and interpreted the data. JS and CS recruited patients and contributed samples. FM, TJ, and LB wrote the manuscript and prepared the figures. All authors reviewed the manuscript.

### Conflict of interest statement

The authors declare that the research was conducted in the absence of any commercial or financial relationships that could be construed as a potential conflict of interest.

## Supplementary material

Supplementary Figure 1 CD160 remains expressed by CD8^+^ T cells, while the proliferation marker Ki67 is reduced to steady-state conditions by d 20 p.i. Lymphocytes from peripheral blood from PbA infected mice were isolated at indicated time points and CD8^+^ T cells were analyzed by flow cytometry for CD160 and Ki67 expression. One of two independent experiments including 3 mice/group is shown.

Supplementary Figure 2 Characterization of CD160^−/−^ mice. **(A)** CD160 amino acid sequence, disulfide bounds, glycosylation sites, the lipidation site and known structural domains are depicted according to uniprot data. For the -/- protein, the amino acid sequence subsequent of the site of deletion was calculated by the ExPASy translation tool. **(B)** Ear tissue lysates were used for amplification of the DNA sequence including the CRISPR/Cas9 target site by PCR. The size of the PCR products was analyzed by agarose gel electrophoresis. An exemplary gel with samples from eight mice (M1-M8, lane 3-10) and a negative control without template (H_2_O, lane 2) is shown. The PCR product size is annotated according to the 500 bp ladder (lane 1). **(B)** PCR products were digested by Bpu10I and the size again analyzed by agarose gel electrophoresis. The genotype referring to the analyzed mice is annotated: +/+ wild type, +/- heterozygous, -/- homozygous knockout. (C,D) WT and CD160^−/−^ mice were infected with PbA and organs were collected at d 6 p.i. CD3^+^ cells from the spleen **(C)** or blood **(D)** were analyzed by flow cytometry for CD160 expression. Representative plots of two independent experiments are shown. **(E)** Intestinal intraepithelial cells from naïve WT and CD160^−/−^ mice were analyzed by flow cytometry for CD160 expression on non-hematopoietic cells (CD8^−^CD45^−^) and hematopoietic cells (CD45^+^), being positive or negative for CD8. Representative plots of two independent experiments are shown. Frequency of T cell subsets (CD4/CD8; TCRβ/γδ), B cells (CD19) and NK cells (NK1.1) within splenocytes **(F)** and CD4/CD8 T cells in the thymus **(G)** was assessed by flow cytometry. Representative plots out of two independent experiments are shown.

Supplementary Figure 3 Parasitemia of HVEM^−/−^ and CD160^−/−^ mice. The frequency of PbA infected RBC at day 6 p.i. of HVEM^−/−^**(A)** or CD160^−/−^ **(B)** mice is shown. Data is pooled from 8 **(A)** or three **(B)** independent experiments including 3–6 mice/group. ^*^*p* < 0.05.

Supplementary Figure 4 Gating strategy for murine cells. Flow cytometry data of murine samples was gated according to the strategy shown.

Supplementary Figure 5 Gating strategy for human cells. Flow cytometry data of human samples was gated according to the strategy shown.
