# Supplementary figures and images for: HVEM and CD160: Regulators of Immunopathology During Malaria Blood-Stage
Source: Front Immunol. 2018 Nov 13;9:2611. doi: 10.3389/fimmu.2018.02611 (PMC6243049; doi:10.3389/fimmu.2018.02611)

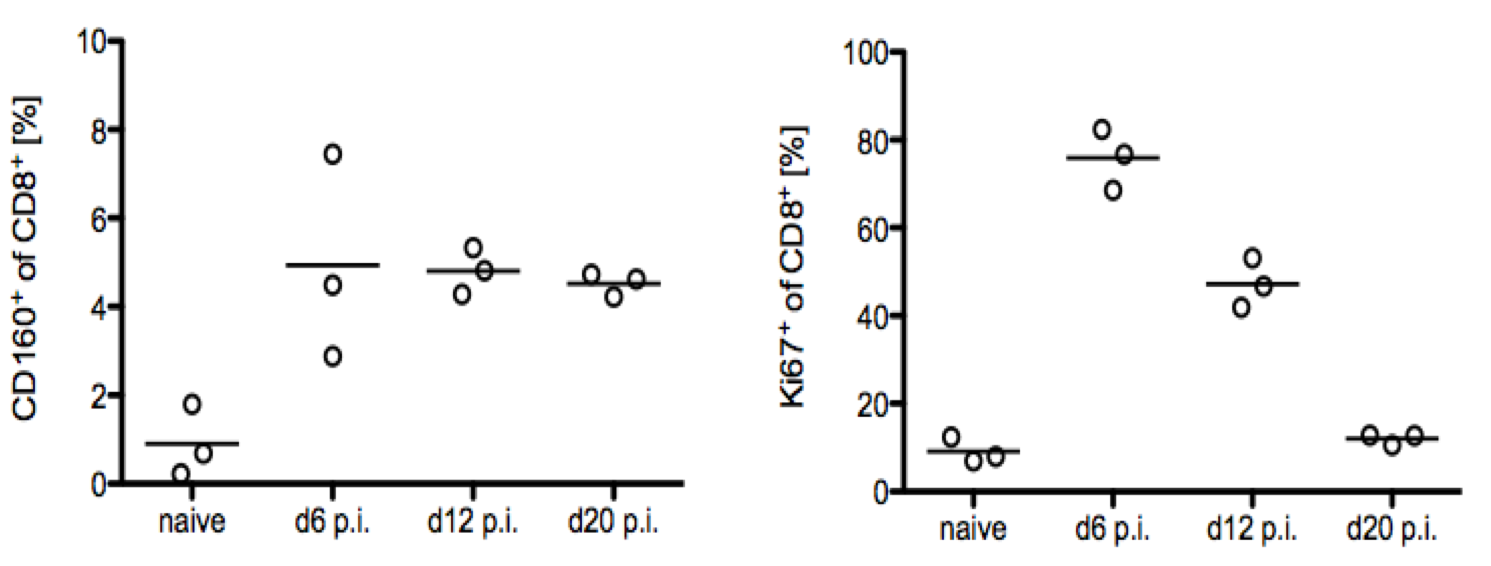

Supplement: Supplementary Figure 1 — CD160 remains expressed by CD8+ T cells, while the proliferation marker Ki67 is reduced to steady-state conditions by d 20 p.i. Lymphocytes from peripheral blood from PbA infected mice were isolated at indicated time points and CD8+ T cells were analyzed by flow cytometry for CD160 and Ki67 expression. One of two independent experiments including 3 mice/group is shown. [file Image_1.TIFF]

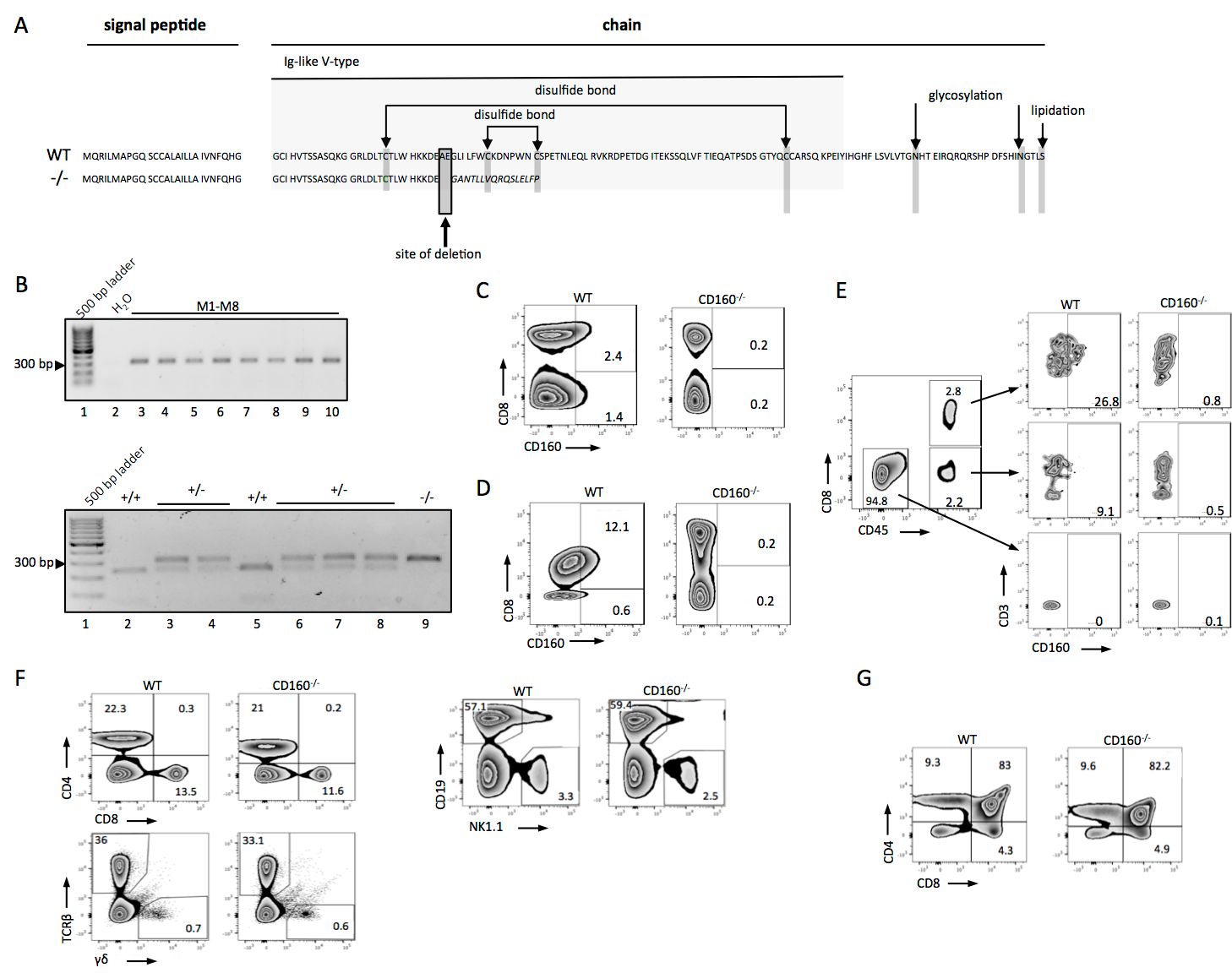

Supplement: Supplementary Figure 2 — Characterization of CD160−/− mice. (A) CD160 amino acid sequence, disulfide bounds, glycosylation sites, the lipidation site and known structural domains are depicted according to uniprot data. For the -/- protein, the amino acid sequence subsequent of the site of deletion was calculated by the ExPASy translation tool. (B) Ear tissue lysates were used for amplification of the DNA sequence including the CRISPR/Cas9 target site by PCR. The size of the PCR products was analyzed by agarose gel electrophoresis. An exemplary gel with samples from eight mice (M1-M8, lane 3-10) and a negative control without template (H2O, lane 2) is shown. The PCR product size is annotated according to the 500 bp ladder (lane 1). (B) PCR products were digested by Bpu10I and the size again analyzed by agarose gel electrophoresis. The genotype referring to the analyzed mice is annotated: +/+ wild type, +/- heterozygous, -/- homozygous knockout. (C,D) WT and CD160−/− mice were infected with PbA and organs were collected at d 6 p.i. CD3+ cells from the spleen (C) or blood (D) were analyzed by flow cytometry for CD160 expression. Representative plots of two independent experiments are shown. (E) Intestinal intraepithelial cells from naïve WT and CD160−/− mice were analyzed by flow cytometry for CD160 expression on non-hematopoietic cells (CD8−CD45−) and hematopoietic cells (CD45+), being positive or negative for CD8. Representative plots of two independent experiments are shown. Frequency of T cell subsets (CD4/CD8; TCRβ/γδ), B cells (CD19) and NK cells (NK1.1) within splenocytes (F) and CD4/CD8 T cells in the thymus (G) was assessed by flow cytometry. Representative plots out of two independent experiments are shown. [file Image_2.TIFF]

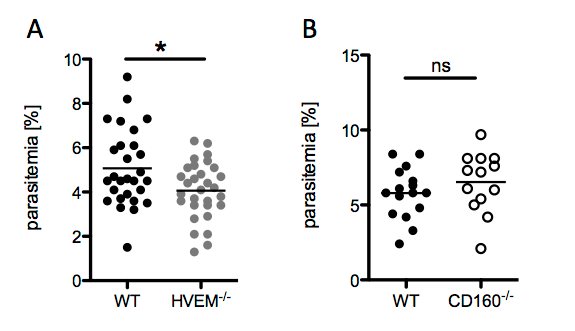

Supplement: Supplementary Figure 3 — Parasitemia of HVEM−/− and CD160−/− mice. The frequency of PbA infected RBC at day 6 p.i. of HVEM−/−(A) or CD160−/− (B) mice is shown. Data is pooled from 8 (A) or three (B) independent experiments including 3–6 mice/group. *p < 0.05. [file Image_3.TIFF]

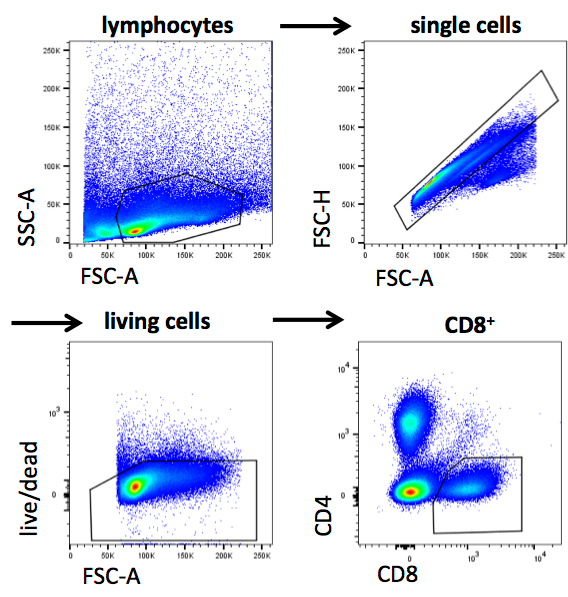

Supplement: Supplementary Figure 4 — Gating strategy for murine cells. Flow cytometry data of murine samples was gated according to the strategy shown. [file Image_4.TIFF]

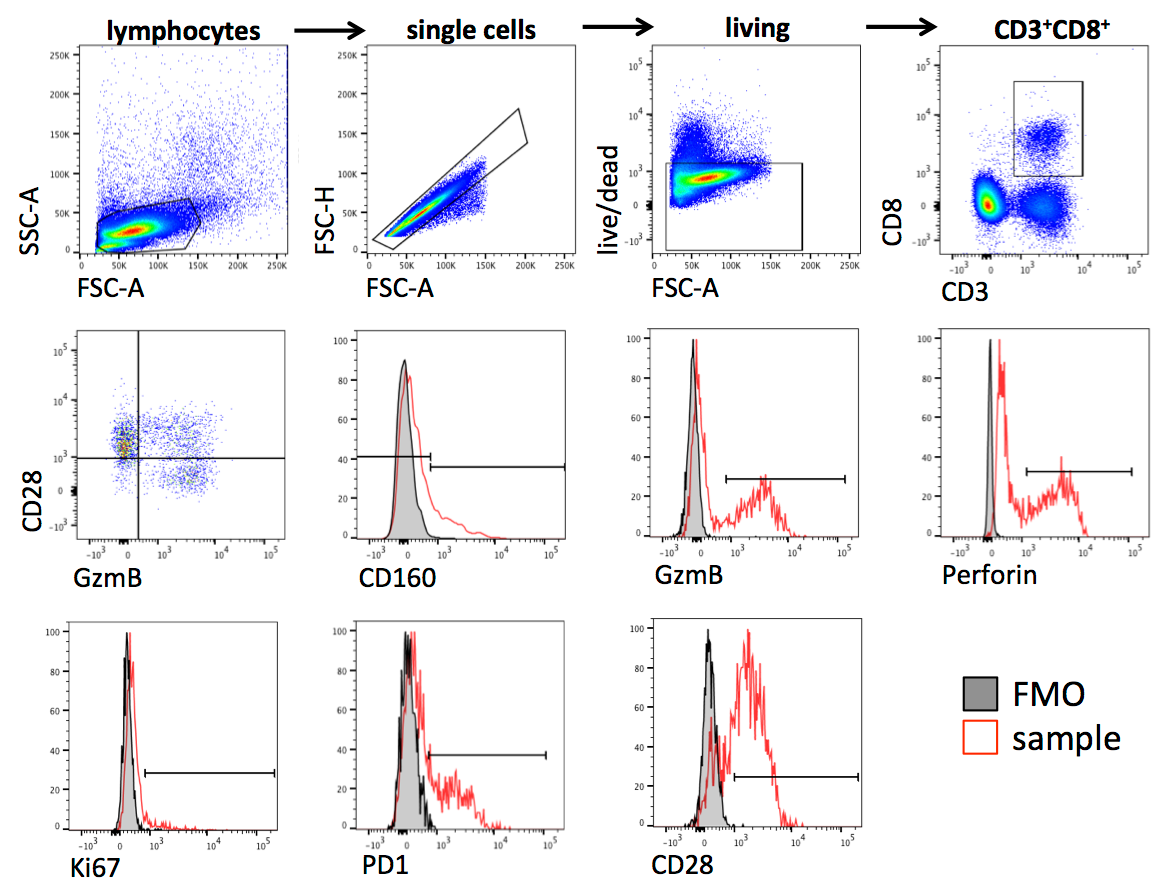

Supplement: Supplementary Figure 5 — Gating strategy for human cells. Flow cytometry data of human samples was gated according to the strategy shown. [file Image_5.TIFF]
